# Autophagy confers DNA damage repair pathways to protect the hematopoietic system from nuclear radiation injury

**DOI:** 10.1038/srep12362

**Published:** 2015-07-21

**Authors:** Weiwei Lin, Na Yuan, Zhen Wang, Yan Cao, Yixuan Fang, Xin Li, Fei Xu, Lin Song, Jian Wang, Han Zhang, Lili Yan, Li Xu, Xiaoying Zhang, Suping Zhang, Jianrong Wang

**Affiliations:** 1 Hematology Center of Cyrus Tang Medical Institute, Jiangsu Institute of Hematology, Collaborative Innovation Center of Hematology, Jiangsu Key Laboratory for Stem Cell Research, Soochow University School of Medicine, Suzhou 215123, China

## Abstract

Autophagy is essentially a metabolic process, but its *in vivo* role in nuclear radioprotection remains unexplored. We observed that *ex vivo* autophagy activation reversed the proliferation inhibition, apoptosis, and DNA damage in irradiated hematopoietic cells. *In vivo* autophagy activation improved bone marrow cellularity following nuclear radiation exposure. In contrast, defective autophagy in the hematopoietic conditional mouse model worsened the hematopoietic injury, reactive oxygen species (ROS) accumulation and DNA damage caused by nuclear radiation exposure. Strikingly, *in vivo* defective autophagy caused an absence or reduction in regulatory proteins critical to both homologous recombination (HR) and non-homologous end joining (NHEJ) DNA damage repair pathways, as well as a failure to induce these proteins in response to nuclear radiation. In contrast, *in vivo* autophagy activation increased most of these proteins in hematopoietic cells. DNA damage assays confirmed the role of *in vivo* autophagy in the resolution of double-stranded DNA breaks in total bone marrow cells as well as bone marrow stem and progenitor cells upon whole body irradiation. Hence, autophagy protects the hematopoietic system against nuclear radiation injury by conferring and intensifying the HR and NHEJ DNA damage repair pathways and by removing ROS and inhibiting apoptosis.

Bone marrow injury is one of the worst consequences of extreme nuclear radiation exposure, such as from nuclear weapons and nuclear accidents^1^,^2^ and is also one of the major limiting factors for radiation therapy for cancer^3^. The risk of carcinogenesis induced by radiation treatment is significantly high for hematopoietic tissue. Exposure to ionizing radiation causes severe oxidative stress and subsequent double-strand breaks (DSBs) in genomic DNA. Unrepaired DNA can lead to mutagenesis and malignant transformation in response to accumulated low radiation exposures, and even organ failure or loss of life upon exposure to high doses of irradiation^4^,^5^. DSBs are primarily removed by HR and NHEJ DNA damage repair mechanisms^6^. HR employs the BRCA1/2-RAD51– or MRE11-RAD50-NBS1–mediated DSB repair pathway, which is active in cycling cells, such as proliferating hematopoietic stem cells (HSCs) and progenitor cells^7^,^8^. In contrast, the NHEJ pathway consists of the DNA-dependent protein kinase catalytic subunit and the Ku80/Ku70 heterodimer, as well as the DNA ligase IV/XRCC4/XLF complex^9^. The NHEJ pathway is believed to be the main mechanism for DSB repair in quiescent cells, such as HSCs^10^,^11^,^12^,^13^,^14^. Although the NHEJ mechanism is considered intrinsically error prone^15^, it does not usually join unlinked DNA ends^16^,^17^.

Autophagy is essentially a cellular metabolic process that removes unnecessary or harmful substances via lysosomal degradation machinery. This process buffers against various stresses, in particular ROS, protects against apoptotic and pathogen insults, and clears damaged organelles^18^,^19^. A recent *in vitro* study indicated that autophagy prevents irradiation injury and maintains stemness by decreasing ROS generation in mesenchymal stem cells^20^. However, the role of autophagy in DNA damage repair in an *in vivo* system in response to nuclear irradiation remains unexplored. In the present study, we show that autophagy is indispensable for nuclear radioprotection in the hematopoietic system and that artificially strengthened autophagy protects the hematopoietic system in irradiated mice by conferring and intensifying DNA damage repair pathways, in addition to removing ROS and inhibiting apoptosis. These findings reveal a new way to protect the hematopoietic system from nuclear radiation exposure.

## Results

### Rapamycin protects *ex vivo* hematopoietic cells against nuclear radiation exposure

**Figure 1:**
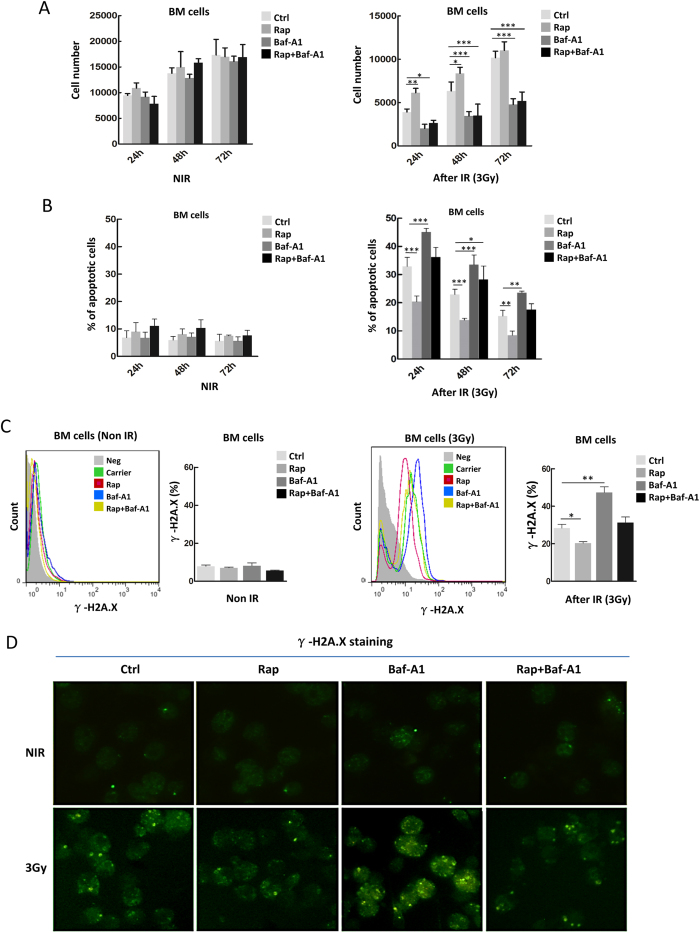
Rapamycin protects *ex vivo* bone marrow cells from radiation damage. (**A**) Rapamycin improves cell proliferation under radiation exposure. Bone marrow cells were isolated from wild-type B6/C57 mice and cultured in Iscove’s Modified Dulbecco’s Media with carrier (DMSO), rapamycin (50 nM), bafilomycin A1 (5 nM), and rapamycin together with bafilomycin A1 for 24 h. After irradiation with 3 Gy, each group of cells was seeded at a density of 5 × 10^3^ cells/well in 96-well culture plates for growth recovery. The cells were counted with a hemocytometer at the indicated postirradiation times. A proliferation advantage was observed in irradiated bone marrow cells pretreated with rapamycin, which was blocked by bafilomycin A1 (right panel). Neither rapamycin nor bafilomycin A1 altered the overall proliferation of total non-irradiated bone marrow cells (left panel). (**B**). Rapamycin inhibited the apoptosis of irradiated bone marrow cells, which was blocked by bafilomycin A1. The above irradiated cells were labeled with propidium iodide and FITC-conjugated annexin V for cytometric analysis. Significantly reduced cell apoptosis was observed in cells treated with rapamycin, and bafilomycin A1 reduced the reduction in apoptosis (right panel). Neither rapamycin nor bafilomycin A1 altered the obvious apoptosis level of total non-irradiated bone marrow cells (left panel). (**C**) Rapamycin reduced the DNA damage of irradiated bone marrow cells, which was reversed by bafilomycin A1 (right panel). Neither of the two drugs altered DNA damage level of the non-irradiated bone marrow cells (left panel). The DNA damage response was measured with specific marker γ-H2A.X in the above irradiated or non-irradiated cells. Shown are a representative flow histogram and statistical data for γH2A.X–positive cells. (**D**) Representative immunofluorescence microscopic data for γH2A.X foci formation in the same groups of bone marrow cells as in C. Data are mean ± SD from at least three independent experiments. n ≥ 5, *p < 0.05 and **p < 0.01.

To explore a possible role for autophagy in protecting the hematopoietic system against nuclear radiation exposure, we first isolated bone marrow cells from mice and treated with or without rapamycin, an autophagy inducer. The results show that rapamycin protected *ex vivo* bone marrow cell proliferation from nuclear irradiation exposure, whereas treatment with bafilomycin A1, an autophagy inhibitor, reduced the rapamycin-induced protection of bone marrow cell proliferation. In contrast, as compared with the carrier DMSO, neither rapamycin nor bafilomycin A1 at the same concentrations caused an obvious decrease in cell proliferation without radiation exposure (Fig. 1A), suggesting that rapamycin or bafilomycin A1 at such concentration does not cause detectable change of overall numbers of bone marrow hematopoietic cells. In line with the above results, autophagy inducer rapamycin reduced the apoptotic death of irradiated bone marrow cells, but autophagy inhibitor bafilomycin A1 increased the apoptosis of bone marrow cells exposed to the radiation, whereas .rapamycin or bafilomycin A1 at the same concentrations neither activate nor inhibit apoptosis of bone marrow cells without nuclear radiation exposure (Fig. 1B). Furthermore, rapamycin decreased the radiation-induced DNA damage of bone marrow cells, and bafilomycin A1 caused the opposite effect, as seen by examination of the DSB DNA damage marker γH2AX by flow cytometry (Fig. 1C, right panel) and confocal microscopy (Fig. 1D, lower panel), whereas the control groups not exposed to the radiation did not show detectable changes in DNA damage response (Fig. 1C left panel, 1D upper panel). The above data from autophagy inducer or inhibitor treatment suggest a possible role of autophagy in *ex vivo* protection of hematopoietic cells.

### Rapamycin protects *in vivo* hematopoietic cells against radiation exposure

**Figure 2:**
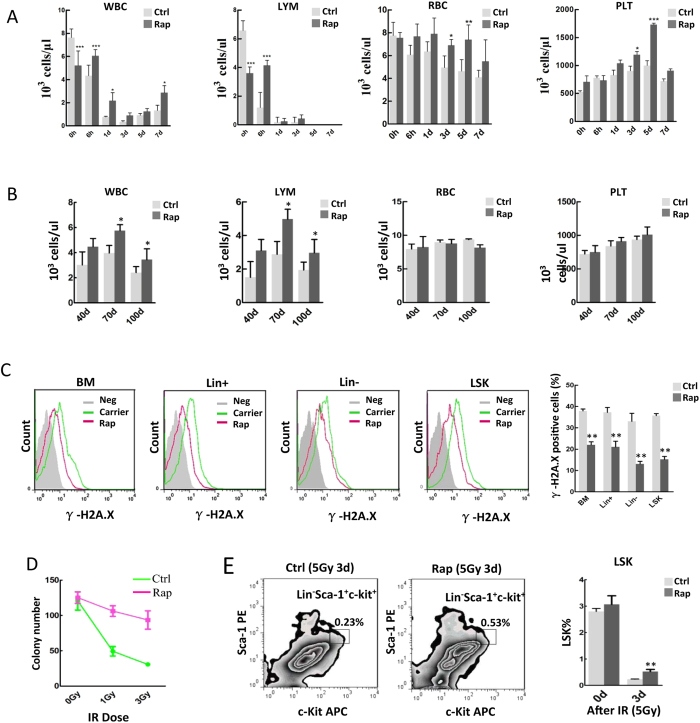
Rapamycin protects *in vivo* hematopoietic cells against radiation exposure (**A,B**) Mice in the treatment group were given 4 mg/kg of rapamycin by i.p. injection five times every other day before total body irradiation, whereas mice in the control group were injected with carrier DMSO. After irradiation, peripheral blood counts were performed at the indicated time points. In the rapamycin-treated group, white blood cell, lymphocyte, red blood cell, and platelet numbers were markedly higher than those of the control group soon after irradiation (**A**), but only white blood cell and lymphocyte counts were higher than those of the control for the long period post-irradiation (**B**). (**C**) Expression of γH2A.X was measured by flow cytometry in total bone marrow cells, lineage-positive cells, lineage-negative cells, and LSK cells of the two mouse groups following 24 h *ex vivo* incubation after whole body irradiation of the mice. DNA damage was significantly reduced in the rapamycin-treated group (right panel). Left panels are representative flow histograms of the treatment groups. (**D**) Rapamycin improved bone marrow progenitors’ colony formation under radiation exposure. Mice were pretreated with carrier or rapamycin for five times before γ-irradiation. Hematopoietic mononuclear cells were then collected from the bone marrow of the sacrifice mice at day 1 after 0 Gy, 1 Gy and 3 Gy by Ficoll gradient centrifugation. Clonogenic progenitors were determined in methylcellulose medium using 6 × 10^3^ bone marrow mononuclear cells per 35 mm dishes and incubated for 7 days. The number of colonies containing more than 50 cells was determined. Colony numbers in the rapamycin-treated group were significantly increased after the radiation exposure. (**E**) LSK cells (Lin^−^Sca-1^+^c-Kit^+^) were sorted with fluorescent-activated cell sorting followed by magnetic-activated cell sorting for lineage-negative cells. Representative LSK sorting gates are shown (left and middle panels). The percentage number of LSK cells, expressed as a percentage against the number of total bone marrow mononuclear cells, was higher in the rapamycin-treated group than in the control group at day 3 after 5 Gy whole body irradiation (right panel). Data are mean ± SD from at least three independent experiments. n ≥ 5, *p < 0.05 and **p < 0.01.

Next, we sought to test a possible *in vivo* radioprotective role on hematopoietic cells by treatment with autophagy inducer. Mice in the rapamycin-treated group were given 4 mg/kg of the drug by intraperitoneal (i.p) injection every other day, five times before nuclear irradiation, whereas mice in the control group were injected with carrier (5% PEG-400, 5% Tween 80). After irradiation, peripheral blood counts were conducted at different time points. Administration of rapamycin protected mouse peripheral blood cells, including white blood cells, lymphocytes, red blood cells and platelets in the short-term post-irradiation period (Fig. 2A). The protective effect remained significant in white blood cells and lymphocytes but not red cells and platelets, for the long-term up to day 100 post-irradiation (Fig. 2B), suggesting that red blood cells and platelets are more vulnerable to radiation exposure, possibly attributed to the facts that both types of the cells lack in nucleus with shorter lifespans and autophagy machinery may not be as effective as those cells with nucleus that is capable of continuous supply with autophagy-essential proteins. Autophagy inducer rapamycin reduced whole body irradiation-caused DNA damage in bone marrow cells, including differentiated hematopoietic cells (Lin^+^), late hematopoietic progenitor cells (Lin^−^), and hematopoietic stem and progenitor cells (Lin^−^Sca-1^+^ c-kit^+^, LSK), as shown by a reduced γH2AX level (Fig. 2C). Hematopoietic progenitor cells isolated from radiation-exposed mice treated with rapamycin retained a relatively high colony-forming ability, whereas the counterpart cells from the whole body irradiated mice without rapamycin treatment displayed a significantly lower colony-forming ability (Fig. 2D). Treatment with rapamycin partially rescued hematopoietic stem and progenitor cells of the mice exposed to nuclear radiation, as seen by a higher number of LSK cells in the rapamycin-treated group on post-irradiation day 3 (Fig. 2E).

### Loss of autophagy in hematopoietic system leads to an elevated radiosensitivity

**Figure 3:**
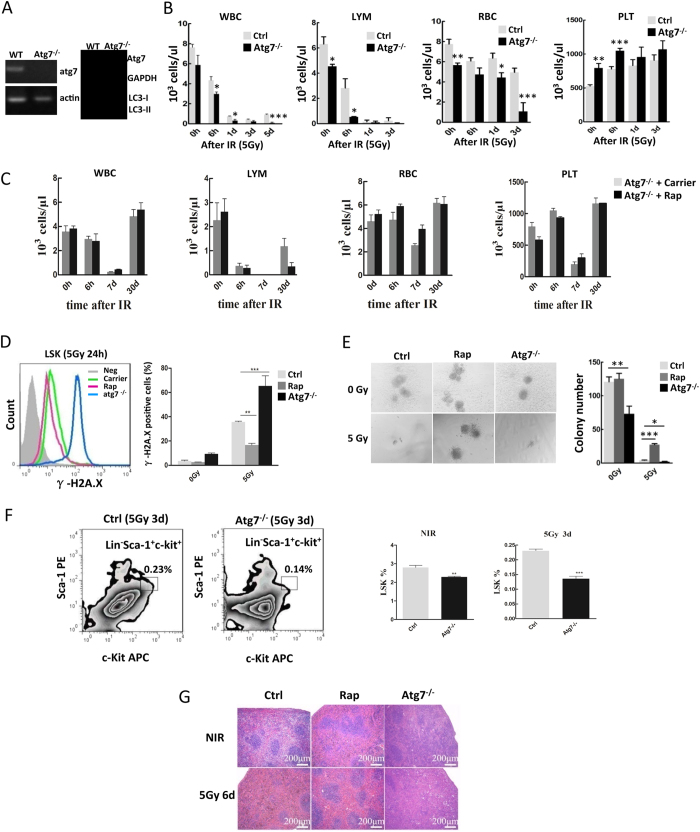
Loss of autophagy in the hematopoietic system leads to an elevated radiosensitivity. (**A**) Induction of autophagy-essential gene atg7 deletion. Atg7^f/f^-Mx1-Cre mice (4 weeks old) were given 1 mg/ml pIpC by i.p. injection five times every other day. Six weeks later, atg7 deficiency in the liver caused hepatomegaly. Atg7 transcripts in bone marrow cells were detected by RT-PCR. Actin cDNA was amplified as an internal control. The protein extracts of bone marrow were immunoblotted with antibodies against Atg7, LC3, and GAPDH. The data show atg7 deficiency and a loss of the autophagy response (no LC3 lipidation) in the bone marrow. (**B**) Loss of autophagy caused reduced peripheral blood counts regardless irradiation. After irradiation, peripheral blood counting was performed at different time points. In the atg7^−/−^ group, the numbers of white blood cells, lymphocytes, and red blood cells, but not platelets, decreased markedly compared with those of the control groups in the short time period. (**C**) Rapamycin failed to rescue the proliferation of atg7^−/−^ bone marrow cells from the mice exposed to radiation. (**D**) Loss of autophagy led to increased DNA damage. Flow cytometric detection of DNA damage marker γH2AX levels in HSPCs from wild-type mice with or without rapamycin treatment, and atg7^−/−^ mice in response to radiation exposure. (**E**) Loss of autophagy reduced colony formation ability regardless of radiation exposure. The number of colonies in the rapamycin group significantly increased after the exposure; conversely, the numbers of colony-forming units in atg7-deficient mice decreased after the exposure. (**F**) Autophagy defect caused less HSPCs under irradiation. There were fewer HSPCs, expressed as the percentage of bone marrow mononuclear cells, in atg7-deficient mice than in the control group. The left and middle panels are representative flow plots and sorting gates. The right panel is the statistic results. (**G**) Morphology of the spleen of C57 mice (treated with carrier or rapamycin) and atg7-deficient mice after 5Gy whole body irradiation. Representative hematoxylin and eosin-stained sections are shown. The scale bar indicates 200 μm. All experiments were repeated at least three times. Data are mean ± SD, n ≥ 6, *p ≤ 0.05, **p ≤ 0.001.

To confirm the above observations from pharmacological intervene, we next examined radiation damage in the hematopoietic system using a conditional autophagy defect mouse model (Atg7^f/f^;Mx1-Cre). Cre-mediated deletion of atg7 by pIpC induction abolishes autophagy in this model, as evidenced by the failure of LC3-I conversion to LC3-II after pIpC induction (Fig. 3A). Loss of autophagy caused greater damage in the short-term post-irradiation period in white blood cells, lymphocytes, red blood cells and, paradoxically, platelets in both of wild type and atg7^−/−^ mice showed a transient increase in number at 6 h post-irradiation (Fig. 3B), suggesting that thrombopoiesis may transiently be enhanced by radiation stress possibly to overcome platelet vulnerability due to its lack of long-lasting supply of autophagy-essential proteins. Importantly, rapamycin no longer rescued hematopoietic cells in autophagy defect mice (Fig. 3C); in contrast to the reduced DNA damage seen after rapamycin treatment in wild-type cells, loss of autophagy in HSPCs (LSKs) greatly increased *in vivo* DNA damage, as seen an increased percentage of γ–H2A.X positive cells (Fig. 3D), suggesting that rapamycin activate autophagy to protect hematopoietic cells from nuclear radiation exposure, and this protection is autophagy-dependent. Unlike what was seen after rapamycin activation of autophagy, loss of autophagy worsened the irradiation-impairment of the colony-forming ability of the cells (Fig. 3E). Compared with the wild-type mice, loss of autophagy reduced the percentage of HSPCs in total bone marrow mononuclear cells, and mice exposed to nuclear radiation led to further reduced percentage of HSPCs in total bone marrow mononuclear cells (Fig. 3F). Nuclear irradiation damaged the vascular sinusoids in the spleen, but rapamycin activation of autophagy maintained the sinusoids; in contrast, loss of autophagy impaired the spleen sinusoids, with the damage worse in autophagy-defective mice (Fig. 3G).

### Autophagy confers and intensifies DNA damage repair pathways

**Figure 4:**
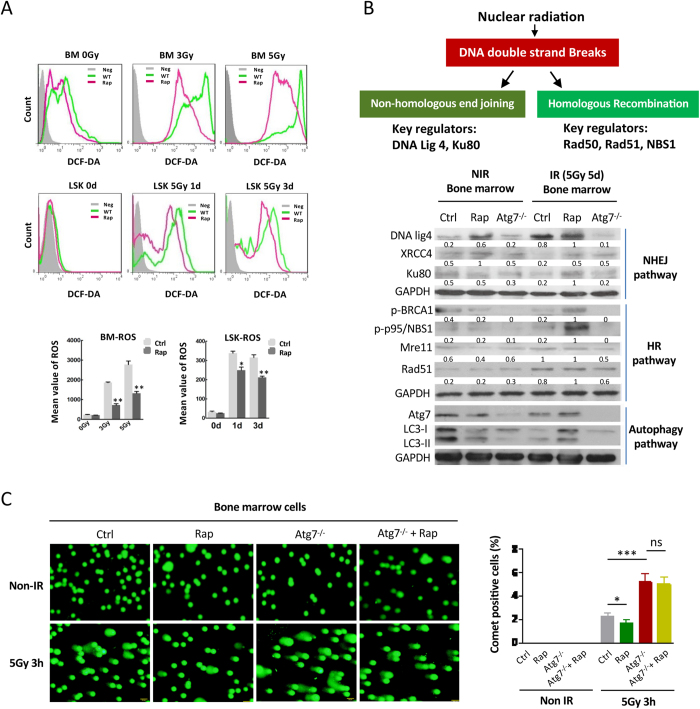
Autophagy confers regulatory proteins required in DSB DNA damage repair. (**A**) Flow cytometric measurement of reactive oxygen species of irradiated bone marrow cells. 2′,7′-dichlorofluorescein diacetate (DCFD) was used as a probe to measure ROS (in particular, this probe detects H_2_O_2_) in the bone marrow or LSKs of control and rapamycin groups by flow cytometry. The cells of the rapamycin group displayed a reduced production of ROS before and after irradiation of mice. (**B**) Immunoblotting of critical proteins in the HR and NHEJ pathways in the bone marrow cell extracts of each group before and after irradiation of mice. (**C**) Comet assay on the bone marrow cells from wild-type or autophagy defective mice with or without radiation exposure. The left panel is representative comet observation with fluorescence microscopy from WT, WT + rapamycin, atg7^−/−^, atg7^−/−^ + rapamycin, and the right panel is the statistical data of the comet assay. Over 1000 cells for each treatment were counted for the percentage of comet positive cells. All dada are from at least three independent experiments. n ≥ 6, *p ≤ 0.05, **p ≤ 0.001.

To understand the radioprotective mechanism of autophagy in the hematopoietic system, we sought to examine ROS levels and DNA damage repair activity in the context of activation or loss of autophagy in mouse hematopoietic system. Activation of autophagy by rapamycin improved ROS clearance in total bone marrow cells and HSPCs from 3Gy or 5Gy irradiation-exposed mice measured on day 1 and 3 post-irradiation with flow cytometric detection of DCF (DCFH-DA) fluorescence, representing ROS oxidative stress (Fig. 4A). The result is consistent with the protective role of autophagy in *ex vivo* mesenchymal stem cells^20^. Rapamycin is also known to reduce ROS via inducing expression of anti-oxidant proteins^21^. It has been known that Atg7^−/−^ mice has elevated ROS accumulation as compared with wild-type mice, and autophagy is the only known mechanism that degrades dysfunctional mitochondria^22^,^23^,^24^, supporting a role of autophagy in removing excessive ROS in physiological condition. It is thus expected that atg7^−/−^ hematopoietic cells after nuclear irradiation accumulates even more ROS. Therefore, the reduction of ROS in hematopoietic cells may be achieved through both autophagy-dependent and autophagy-independent pathways.

Mitigation of nuclear radiation injury largely depends on double-stranded DNA damage repair mechanisms. Mice lacking components of the DNA damage response and DSB repair mechanisms all displayed severe hematopoietic phenotypes and HSC defects^12^,^13^,^25^. We thus examined whether activation or loss of *in vivo* autophagy causes an alteration in double-stranded DNA damage repair pathways. The result showed that for NHEJ pathway, activation of autophagy by rapamycin significantly induced the expression of DNA ligase 4, regardless of radiation exposure. In wild-type mice, the DNA ligase 4 expression in hematopoietic system can also be strongly induced upon radiation exposure, but loss of autophagy no longer caused DNA ligase 4 induction in the hematopoietic system of the Atg7^−/−^ mice, suggesting that DNA ligase 4 induction responsive to nuclear radiation strongly depends on autophagy. XRCC4 and Ku 80, two other members of NHEJ pathway, were also slightly increased upon rapamycin treatment, and in particular, loss of autophagy caused a failed induction of Ku 80 upon nuclear irradiation (Fig. 4B). For HR pathway, upon nuclear radiation exposure, activation of autophagy with rapamycin upregulated phosphorylated BRCA1 and phosphorylated p95/NBS1, whereas loss of autophagy caused loss of the two phosphorylated proteins (Fig. 4B), suggesting that maintaining and/or activation of these two proteins depends on autophagy machinery. The above data also reveal that DNA ligase 4 (in NHEJ pathway) and p95/NBS1 (in HR pathway) may be the major players in double-stranded DNA damage repair mechanisms in mononuclear hematopoietic cells under nuclear radiation stress, and both of which critically depends on an intact autophagy machinery in hematopoietic system. Rad 51 and Mre11, however, appeared not to be regulated by rapamycin or autophagy mechanism (Fig. 4B). Similarly, activation of autophagy by rapamycin or loss of autophagy in atg7^−/−^ mice did not alter or induce protein levels of Rad 50, p53 and PTEN (data not shown), which have been known implicated in DNA damage repair in other cell-type or stress contexts^7^,^8^,^9^,^10^. The above data thus support our notion that the DNA damage repair proteins including DNA ligase 4, Ku 80, XRCC4, BRCA1 and p95/NBS1 in hematopoietic system are specifically regulated by autophagy upon nuclear radiation exposure.

To further support our contention that autophagy mitigates nuclear radiation injury via sustaining DNA damage repair proteins, we performed a comet assay on the bone marrow cells of the mice exposed to nuclear radiation. The results showed that under non-irradiation condition, there was no detectable comet tail shape in the bone marrow cells of wild-type or atg7^−/−^ mice, treated with or without rapamycin (Fig. 4C); in contrast, loss of autophagy by atg7 deletion caused significantly elevated percentage of comet positive bone marrow cells, and rapamycin reduced the percentage of comet positive bone marrow cells in wild type mice, but failed to do so in atg7^−/−^ mice (Fig. 4C), thereby concluding that autophagy protects against nuclear radiation injury via conferring and intensifying DNA damage repair pathways.

## Dicussion

Hematological targets of nuclear radiation damage are of great significance when protecting against normal tissue injury. Only limited protective options are currently available for radiation therapy and radiological or nuclear accidents/attacks. Generation of excessive ROS and induction of apoptosis by the ionization of irradiated matter are major causes of tissue injury from nuclear radiation. Thus, clearance of ROS and the intervening apoptosis pathway have been two major strategies for radioprotection, and a well-characterized radioprotection strategy involves using antioxidants, which act as scavengers of ROS. Abdel-Mageed and colleagues reported that intravenous administration of mesenchymal stem cells, which were genetically modified to overexpress extracellular superoxide dismutase, efficiently scavenger ROS and improve the survival of irradiated mice^26^. Some chemicals or compounds also have radioprotective power. For instance, alpha-asarone prevents genotoxicity and hematopoietic injury in mammalian organisms^27^. Ferulic acid improves hematopoietic cell recovery in whole-body gamma irradiated mice^28^. L-arginine improves radioprotection for hematopoietic progenitor cells^29^. CpG-oligodeoxynucleotide, a synthetic analog of bacterial DNA, minimizes the bone marrow damage induced by total body irradiation^30^. Antioxidant dietary supplementation in mice exposed to proton radiation attenuates expression of programmed cell death-associated genes^31^.

In radiation therapy of cancer, selective protection of normal cells via suppression of apoptosis, for instance by pharmacological inhibition of p53 or activation of NF-κB, is more important. The p53 inhibitors pifithrins show radioprotective efficacy in mice^32^. Furthermore, fine-tuning p53 activity through C-terminal modification significantly improves HSC homeostasis and mouse radiosensitivity^33^. NF-κB is generally inactive in normal cells, but it is constitutively activate in most tumors^34^. NF-κB drives expression of inflammatory cytokines, which have a long-recognized radioprotective effect^35^; however, unacceptable toxicity has prevented their clinical development as radioprotectants. Nonetheless, an NF-κB–activating approach to radioprotection has been proven feasible by the demonstration that bacterial flagellin, which is an agonist of TLR5 and a natural NF-κB–activating agent, has outstanding radioprotective properties in mice and primates^36^. Pharmacologically constitutive engagement of NF-κB activity specifically protects normal cells in mouse tumor models^36^. Similarly, administration of EGF promotes the recovery of the HSC pool *in vivo* and improves the survival of mice after total body irradiation. EGF reduces radiation-induced apoptosis of HSCs, mediates through repression of the proapoptotic protein PUMA^37^. Synthetic lipopeptide agonists of Toll-like receptor 2 prevent and mitigate acute radiation syndrome in mice, also by targeting the apoptosis pathway^38^.

An alternative radioprotection strategy is to induce reversible normal cell cycle arrest at the G1/S transition by pharmacological inhibitors of cyclin-dependent kinases, such as PD0332991^39^. PD0332991 has a beneficial effect on the recovery of all peripheral blood lineages: platelets, erythrocytes, myeloid cells, and peripheral blood lymphocytes. Moreover, no sign of myeloproliferative disorder or myelodysplasia was found in animals of long-term surviving cohorts^39^. In addition, blockade of CD47, a receptor for the secreted protein thrombospondin-1, provides local radioprotection of soft tissues and bone marrow. Suppression of CD47 using an antisense morpholino increases the survival of mice exposed to lethal total body irradiation, and increased survival is associated with recovery of hematopoiesis^40^.

In contrast to the previous radioprotection strategies that targeted single pathways, here we show that manipulation of autophagy has comprehensive beneficial effects, not only helping to eliminate ROS and inhibit apoptosis, but also enhancing DNA damage repair mechanisms under radiation stress. Insufficient autophagy due to monoallelic deletion of autophagy-essential gene *Beclin*1 was previously found to cause genome instability in mammary tumorigenesis^41^, and loss of autophagy either by deletion of other autophagy-essential genes *FIP*200 or *atg*7 in mouse embryonic fibroblast cells impairs DNA damage repair^42^,^43^, both revealing a connection between *in vitro* autophagy and DNA damage repair. We observed here that *ex vivo* autophagy activation reversed the irradiation-induced proliferation inhibition, apoptosis, and DNA damage of hematopoietic cells. *In vivo* autophagy activation improved bone marrow cellularity caused by nuclear radiation exposure. On the contrary, defective autophagy in a hematopoietic conditional mouse model worsened hematopoietic injury, ROS clearance, and DNA damage in response to nuclear radiation, indicating that activation of autophagy not only inhibits apoptosis, but also promotes ROS scavenging. Surprisingly, defective autophagy caused an absence of or a reduction in an extensive array of regulatory proteins critical to both HR and NHEJ DNA damage repair pathways, as well as a failure to induce these proteins in response to nuclear radiation, whereas activation of autophagy increased most of these proteins tested in hematopoietic cells. Jasin and Gorbunova’s groups have developed plasmid-based assays to directly assess whether HR and NHEJ pathways are involved in DNA damage response^44^,^45^,^46^. Unfortunately, the transfection of these plasmids into mouse hematopoietic primary cells proved to be too low efficiency to reach a meaningful result in our attempt. However, comet assay under neutral condition, which primarily detects double-stranded DNA breaks^47^, indicated that loss of autophagy caused increased DSBs and activation of autophagy reduced DSBs in wild-type cells but not autophagy defect cells (Fig. 4C). Thus, the comet assay under neutral condition provides a supportive evidence that autophagy mitigates nuclear radiation injury through regulating the DSB repair pathways.

The increase in DNA damage repair proteins through autophagy, however, seems to be contradictory to the established role of autophagy in degradation of proteins. The data presented in our study suggests that autophagy may regulate DNA damage repair proteins through an indirect way, for instance, through degradation of certain proteins that inhibit the expression of the DNA damage repair proteins, or through inhibition on ubiquitin-proteasomal degradation of the DNA damage repair proteins. Mechanistic study on nuclear radioprotection of hematopoietic system by autophagy, in particular identification of the direct target of autophagy in response to nuclear radiation exposure will be our next pursuit.

## Methods

### Animals

C57BL/6 male mice (8–10 weeks old) were treated rapamycin (Calbiochem, Darmstadt, Germany)-or carrier (5% PEG-400, 5% Tween 80). Each group had at least 20 mice and some mice of each group were sacrificed at the indicated times. Conditional mouse model Atg7^f/f^;Mx1-Cre was generated from crossing Atg7^f/f^ mice^42^ with MX1-Cre mice (from Jackson Lab, USA). Briefly, rapamycin was reconstituted in absolute ethanol at 10 mg/ml and diluted in 5% Tween-80 and 5% PEG-400. Mice in the Rap-treated group were given 4 mg/kg rapamycin by i.p injection every other day, five times before irradiation, whereas mice in the control group were injected with carrier (5% PEG-400, 5% Tween 80). Half of the mice in each group were irradiated over their entire bodies with 3 or 5 Gy from ^60^Co γ-ray irradiation with a dose rate of 1Gy/min. Blood sample was taken from mouse caudal vein. On the first, third, and fifth days after irradiation, mice were sacrificed. All experiments with mice were complied with the institution’s regulations on animal welfare protocols and were approved by the university’s ethics committee of laboratory animals.

### Isolation of mouse bone marrow cells and hematopoietic stem and progenitor cells and *ex vivo* treatment of rapamycin and bafilomycin A1

To obtain total bone marrow cells, whole femurs were isolated by removing the attached muscles rapidly. The bone marrow was flushed with PBS using a syringe with a 25-gauge needle. Bone marrow aspirates were subjected to Ficoll Paque gradient centrifugation (Amersham Pharmacia Biotech, Piscataway, USA), and mononuclear cells (MNCs) were separated. MNCs were placed in IMDM (Sigma, USA) containing 20% fetal bovine serum, 2 mM L-glutamine, 100 U/mL penicillin, and 100 U/mL streptomycin (Gibco, USA). For the experiments, MNCs were incubated in 96-well culture plates (approximately 5 × 10^3^ BMNCs/well) for 24 to 72 hours. MNCs cells were incubated with media alone or with different concentrations of DMSO (control), rapamycin (100 nM), or bafilomycin A1 (5 nM) for 24 hours at 37 °C. For sorting for hematopoietic stem and progenitor cells, Lineage negative cells were harvested by MACS according to protocol of mouse lineage depletion kit (Miltenyi Biotec, Italy), followed by sorting for HSPCs and HSCs (LSK, and LSKCD34 markers) with FACS (BD Ari III, USA). The subsequent HSPCs or HSCs cells were either analyzed with flow cytometry or transiently cultured in IMDM containing 20% FBS (Hyclon, USA), 100 ng/mL mouse-IL-6, 100 ng/mL mouse Flt-3, 100 ng/mL mouse SCF, 10 ng/mL mouse IL-11 (PeproTech, Rocky Hill, USA).

### Apoptosis analysis

The Annexin V assay was performed with Alexa Fluor 488 Annexin V /Dead cell Apoptosis Kit according to the manufacturer’s instructions (Invitrogen, USA).

### DNA damage assay

To asses DNA damage accumulation and repair capacities, γH2AX-foci were examined before and after exposure to ionizing irradiation with flow cytometry. Comet assay was performed by use of Trevigen’s reagent kit for signle cell gel electrophoresis assay according to the manufacturer’s protocol (Trevigen, USA, Cat# 4250-050-K).

### Immunofluorescence

Immunofluorescent staining was done in accordance with the manufacturer’s protocol. Briefly, cells were fixed in 4% formaldehyde for 10 minutes at 37 °C and immersed in cold methanol for 30 minutes at 4 °C. After blocking, cells were incubated 1 hour at room temperature with anti-γH2AX rabbit mAb (1:100, Alexa 488). The antibodies were from Cell Signaling Technology, Danvers, USA. Excess of unbound antibody was removed at each step by three washes with PBS. The images were obtained by using an Olympus confocal microscope (FV1000MPE, Olympus, Tokyo, Japan).

### Hematopoietic stem and progenitor cell colony-forming unit (HSPC-CFU) assay

The bone marrow cells were subjected to Ficoll gradient centrifugation to isolate the hematopoietic mononuclear cells from mouse bone marrow. Cells were centrifuged at 400 g for 30 min and the intermediate layer was collected. Clonogenic progenitors were determined in methylcellulose medium (Stem Cell Technologies, Alameda, USA) using 6 × 10^3^ bone marrow mononuclear cells per dishes (35 mm) and incubated in a humidified atmosphere with 5% CO_2_ at 37 °C for 7 days. The number of colonies containing more than 50 cells was determined.

### Blood routine examination

20 μl mouse peripheral blood was added into 500 ul CPK-303A solution (37 °C), and then performed blood routine examination using Sysmex KX-21N (Holliston, USA).

### Measurement of ROS generation

ROS generation was measured as described by Marchetti *et al.*^41^ Briefly, cells (5 × 10^6^/mL) were exposed to 3 or 5Gy radiation. After exposure, cells were incubated in 10 nm 2,7-dichlorofluorescein diacetate (DCFH-DA) (Molecular Probes, USA) at 37 °C for 20 minutes to measure ROS level. The cells were harvested and washed with cold PBS solution 3 times, and the ROS level was determined by fluorescence-activated cell sorter (FACS) analysis.

### Fluorescence Microscopy

The cells were pelleted by centrifugation for 5 minutes at 200 g, washed in PBS, fixed in 4% paraformaldehyde at room temperature for 30 min. After brief rinsing in PBS, the cells were blocked with 5% goat serum in PBS (with 0.3% Triton X-100) for 1 h, then incubated with the primary antibody (diluted in PBS supplemented with 0.3% Triton X-100 and 1% bovine serum albumin, overnight in a humid chamber at 4 °C) followed by incubation with FITC-conjugated secondary antibodies (diluted 1:1000, for 1 h at RT. Excess of unbound antibody was removed at each step by three washes with PBS. The nuclear material was stained with 20 μg/ml Hoechst 33342 (Invitrogen) at RT for 10 min. After a brief washing with PBS, the images were obtained using an Olympus confocal microscope (Olympus, Japan).

### Western blotting analysis

The bone marrow cells from the mouse femur were extracted using lysis buffer (Cell Signaling Technology, Danvers, MA) at the indicated times. The protein concentration of each sample was measured using the BCA reagent (Pierce, Rockford, IL). Equal amounts of proteins were fractionated via 7–15% sodium dodecyl sulfate–polyacrylamide gel electrophoresis (SDS–PAGE) and transferred onto nitrocellulose membranes. Membranes were blocked with 5% non-fat dry milk in TBST (0.2% Tween in TBS) for 1 h and then incubated overnight with antibodies specific to DNA ligase 4, Ku 80, Rad 50, Rad 51, Mre 11 and p-p95/NBS1 (Abcom, USA), XRCC4, p-BRCA1, phospho-P53, P53, phspho-PTEN, PTEN, LC3, ATG7 and GAPDH (Cell Signaling Technology, USA). After three washes with TBST, the blots were probed with the appropriate HRP-conjugated secondary antibodies (Santa Cruz Biotechnology, USA) for 1 h at RT. Detection of the blots was performed using ECL reagents (Amersham Pharmacia Biotechnology, Buckinghamshire, UK), and X-ray films (AGFA, Mortsel, Belgium).

### Statistical analysis

Results are shown as mean ± SD of the data from at least three independent experiments. For statistical comparison between groups, the two-way ANOVA and one-way ANOVA were used, with a p value less than 0.05 considered significant.

## Additional Information

**How to cite this article**: Lin, W. *et al.* Autophagy confers DNA damage repair pathways to protect the hematopoietic system from nuclear radiation injury. *Sci. Rep.* **5**, 12362; doi: 10.1038/srep12362 (2015).
