# Corrigendum: Autophagy confers DNA damage repair pathways to protect the hematopoietic system from nuclear radiation injury

**DOI:** 10.1038/srep30095

**Published:** 2016-07-20

**Authors:** Weiwei Lin, Na Yuan, Zhen Wang, Yan Cao, Yixuan Fang, Xin Li, Fei Xu, Lin Song, Jian Wang, Han Zhang, Lili Yan, Li Xu, Xiaoying Zhang, Suping Zhang, Jianrong Wang

Scientific Reports 5: Article number: 1236210.1038/srep12362; published online: 07212015; updated: 07202016

In this Article, an additional affiliation for Weiwei Lin was omitted. The correct affiliation is listed below:

Department of Histology and Embryology, Medical College, Natong University, 19 Qixiu Road, Nantong 226001, China.

